# A simplified universal approach to COH protocol for IVF: ultrashort flare GnRH-agonist/GnRH-antagonist protocol with tailored mode and timing of final follicular maturation

**DOI:** 10.1186/s13048-015-0198-3

**Published:** 2015-11-04

**Authors:** Raoul Orvieto

**Affiliations:** Infertility and IVF Unit, Department of Obstetrics and Gynecology, Chaim Sheba Medical Center (Tel Hashomer), Ramat Gan, 52621 Israel; Sackler Faculty of Medicine, Tel Aviv University, Tel Aviv, Israel

**Keywords:** Ultrashort flare GnRHa/GnRHant, hCG, GnRH agonist, Ovulation, Trigger, OHSS, Controlled ovarian hyperstimulation, Oocyte quality

## Abstract

Recently, several new promising modifications have been introduced to clinical practice that may simplify and optimize IVF outcome. In the present opinion paper we present a simplified approach to controlled ovarian hyperstimulation protocol (COH), which combines the benefits of the ultrashort flare GnRH agonist/GnRH antagonist protocol and the personalized tailored mode and timing of ovulation triggering, aiming to improve IVF outcome while eliminating of severe OHSS.

In patients at risk to develop severe ovarian hyperstimulation syndrome (OHSS), GnRH agonist (GnRHa) trigger if offered for final follicular maturation. While in those achieving ≥20 oocytes, the freeze all policy with the subsequent frozen-thawed embryo transfers (ET) is recommended, in those where less than 20 oocytes are retrieved, patients are re-evaluated 3 days after oocyte retrieval (day of ET) for signs of early moderate OHSS. If no early signs of OHSS developed, one embryo was transferred, and the patients are instructed to inject 1500 IU of HCG. In cases where signs of early moderate OHSS appear, the freeze all policy is recommended.

In Patients not at risk to develop severe OHSS- three different modes of concomitant administration of both GnRHa and a standard bolus of hCG (5000–10,000 units) prior to oocyte retrieval were suggested. Standard hCG dose concomitant with GnRHa (dual trigger), 35–37 h before oocyte retrieval is offered to normal responders patients, resulting in improved oocyte/embryo quality and IVF outcome. GnRHa 40 h and standard hCG added 34 h prior to oocyte retrieval (double trigger), respectively are offered to patients demonstrating abnormal final follicular maturation despite normal response to COH. The double trigger results in significantly higher number of oocytes retrieved, higher proportions of the number of oocytes retrieved to the number of follicles >10 mm and >14 mm in diameter on day of hCG administration, higher number of MII oocytes and proportion of MII oocytes per number of oocytes retrieved, with the consequent significantly increased number of top-quality embryos, as compared to the hCG-only trigger cycles. Standard hCG dose concomitant with GnRHa (dual trigger), 34 h before oocyte retrieval should be offered to poor responders patients, aiming to overcome premature luteinization, while achieving high yield of mature oocytes.

Further studies are required to support this new concept prior to its implementation as a universal COH protocol to IVF practice.

## Background

Controlled ovarian hyperstimulation (COH) is considered a key factor in the success of in vitro fertilization-embryo transfer (IVF-ET) because it enables the recruitment of multiple healthy fertilizable oocytes and, thereby, multiple as opposed to single ET. COH usually includes the co-administration of gonadotropins and gonadotropin-releasing hormone (GnRH) analogues; the two most commonly used protocols are the long GnRH-agonist (GnRHa) suppressive protocol and the multiple-dose GnRH-antagonist (GnRHant) COH protocol. While the advantages of using GnRH-ant, as opposed to agonists include, mainly, a reduction in the incidence of severe ovarian hyperstimulation syndrome (OHSS) [1], when comparing pregnancy rates, the literature yields conflicting results [2]. In addition, programming of GnRHant cycles continues to be a challenge, and the use of combined oral contraceptives (COCs) pretreatment, which aims to achieve a better synchronized response and a scheduled cycle, was associated with significantly lower ongoing pregnancy rate, longer duration of the stimulation and higher gonadotropin consumption [3].

Recently, several new promising modifications have been introduced to clinical practice, of which, the ultrashort flare GnRHa/GnRHant protocol and the different mode and timing of hCG and GnRHa co-administration for final follicular maturation, have the most prominent impact on IVF outcome.

**Fig. 1:**
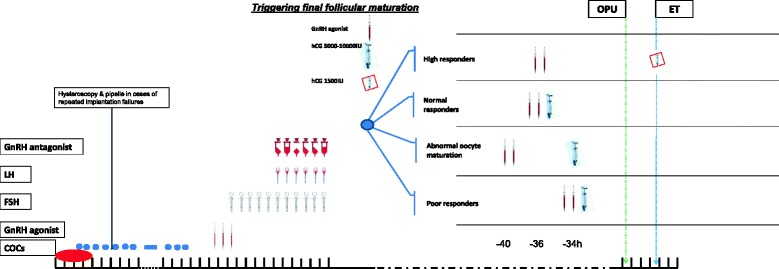
A simplified approach/algorithm to COH protocol, which combines the ultrashort flare GnRHa/GnRHant protocol and the personalized tailored timing of ovulation triggering

Prompted by the aforementioned observations, in our center, conducting up to 1200 IVF cycles per year, we have started to implement a simplified approached to COH protocol. The present opinion paper aims to present this simplified approach (Fig. 1), which combines the benefits of the ultrashort flare GnRHa/GnRHant protocol and the personalized tailored mode and timing of ovulation triggering. We believe that its universal implementation to IVF practice will result in improved outcome while allowing the elimination of severe OHSS.

### The ultrashort flare GnRHa/GnRHant protocol

The ultrashort flare GnRHa/GnRHant protocol was recently introduced to the COH protocols armamentarium [4]. It offers all the advantages of using GnRHant, including a lack of hypoestrogenism, shorter treatment duration and lower gonadotropin requirement. Moreover, it allows cycle programming and offers successful outcome in a variety of challenging cases such as poor responders, and patients with poor embryo quality or repeated IVF failures [5–7]. In addition, this protocol provides protection from severe OHSS by maintaining the option to substitute hCG with GnRHa for final follicular maturation in patients at risk of OHSS [8].

The protocol is comprised of the administration of COCs started on days 2–5 of the menses continued for at least 7 days. GnRHa (e.g. triptorelin 0.1 mg/day) is commenced 3 days after the cessation of the COCs, followed by gonadotropins (FSH only preparations) initiated two days later. GnRHant is added according to the individual program policy (fixed or flexible), and continued until the day of triggering final oocytes maturation. On the day of GnRHant initiation, LH is added to the FSH only preparations.

Final follicular maturation is triggered as soon as the majority of the leading preovulatory follicles have reached a diameter of 17 mm, preferably with a ratio of E2 level to number of leading preovulatory follicles of >14 mm calculated to be lower than 100 pg/mL [9]. All patients should receive luteal support with progesterone starting on the day following oocyte retrieval.

### Triggering final follicular maturation

Recently, different modes and timing of ovulation triggering, using GnRHa, hCG, or both, were adopted to clinical practice [10], attempting to either rescuing the luteal phase and improving reproductive outcome in patients at risk to develop severe OHSS, or improving oocyte/embryo yield and quality in the general IVF population or in patients with abnormal final follicular maturation.

COH using GnRHa trigger has become a common tool aiming to eliminate severe early OHSS and to support the concept of an OHSS-free clinic. However, due to the reported significantly reduced clinical, efforts have been made to improve reproductive outcome. Moreover, while comparing the effect of hCG versus GnRHa trigger on the different follicular maturation variables following an IVF treatment cycle, studies have revealed that the number of oocytes retrieved, percentage of mature oocytes and number of top-quality embryos were either comparable or in favor of the GnRHa trigger.

These advantages were related to the concomitant midcycle FSH peak, observed following GnRHa trigger. The later ensures an adequate complement of LH receptors on the granulosa layer and the synthesis of hyaluronic acid matrix that facilitates the expansion and dispersion of the cumulus cells, allowing the oocyte-cumulus cell mass to become free-floating in the antral fluid [11]. Moreover, while studying the downstream effects of LH receptor activation by LH or hCG, it was demonstrated that LH has a greater impact on AKT and extracellular signal regulated protein kinase (ERK1/2) phosphorylation, responsible for granulosa cells proliferation, differentiation and survival, while hCG generates higher intracellular cAMP accumulation, which stimulates steroidogenesis (progesterone production) [12].

Following the aforementioned observations, GnRHa combined with hCG trigger- for final follicular maturation has been implemented to clinical practice, and the different modes and timing of administration should be appropriately tailored to various subgroup of IVF patients.

### The rationale behind the different stages of the suggested universal protocol

#### COC- The Ultrashort flare

The ultrashort flare GnRHa/GnRHant protocol is comprised of the administration of COCs started on days 2–5 of the menses continued for at least 7 days. COCs pretreatment was shown to result in a better synchronized response and a scheduled cycle on one hand, with significantly longer duration of the stimulation, higher gonadotropin consumption and a questionable lower ongoing pregnancy rate [3, 13, 14]. The detrimental effect of pretreatment COCs in the GnRH antagonist protocol was related to the potential negative effect of the gestagen component on the endometrium, or the low endogenous LH levels induced by COCs, with their deleterious impact on oocyte competence or endometrial receptivity [13].

Regarding the detrimental effects of the gestagen component of the COCs. Recently, the use of anti-androgenic COCs (drospirenone, cyproterone acetate) in oocytes donors was demonstrated to result in a significantly higher oocytes yield, as compared to those using androgenic COCs, and a comparable yield to donors with no COCs pre-treatment. Differences that were maintained also after adjustments for the donor age and total FSH dose used in ovulation induction [15].

#### Repeated IVF failures- When applied to

When applied to patients with repeated IVF failures, the combination of diagnostic hysteroscopy and endometrial sampling during COCs treatment, which precedes the ultrashort GnRH-ag/GnRH-ant protocol, should be offered, resulting in an improved outcome with high implantation and clinical pregnancy rates (42 % as compared to 25 % in patients’ previous conventional IVF cycle) [7].

The ultrashort flare GnRH-agonist stimulates an early follicular phase endogenous FSH release without the concomitant deleterious rises in androgen levels or corpus luteum rescue [16], which is fundamental for follicular recruitment. Moreover, the pretreatment with OCPs was recently shown to suppress pre-GnRH-agonist FSH without blunting the FSH flare and therefore should not affect the follicular response to the GnRH agonist flare. Furthermore, since COC pretreatment blunts the LH flare, thus preventing the detrimental effect of GnRH-agonist induced early rise in follicular androgens with the consequent increase in serum progesterone levels [17], COC should be offered to all patients undergoing the GnRH-agonist flare protocol.

The combination of COCs pretreatment COCs with the microdose flare protocol, which has been offered to poor-responder patients for almost three decades, demonstrated similar results when compared with various other COH protocols, with improved cycle parameters and decreased cancellation rates [16]. This probably results from the flare effect which overcomes the endogenous gonadotropins suppression by the COCs and the aforementioned consequent deleterious impact on oocyte competence or endometrial receptivity.

The GnRH-antagonist provides immediate LH suppression with the possible improvement of the quality of the blastocysts generated [18].

Triggering final follicular maturation by GnRH-agonist, in patients at high risk to develop severe OHSS undergoing the ultrashort GnRH ag/GnRH ant protocol, was shown to be feasible option [8]. The 3 consecutive doses of daily GnRH-a agonist administration at the beginning of ultrashort GnRH-ag/GnRH-ant COH protocol, did not interfere with the ability of the GnRH-agonist to trigger final oocytes maturation at the end of the COH cycle and did not compromise IVF outcome [8, 19].

The aforementioned observation implies that in patients undergoing COH using the ultrashort GnRH-ag/GnRH-ant COH protocol, final follicular maturation may be triggered with hCG, GnRHa or both.

### A simplified approach/algorithm to COH protocol (Fig. 1)

Patients at risk to develop severe OHSS, those with rapidly rising serum E2 levels; peak E2 level in excess of 2500 pg/mL; and/or the emergence of a large number of intermediate sized follicles [20], GnRHa trigger if offered for final follicular maturation. In those achieving ≥20 oocytes, the freeze all policy with the subsequent frozen-thawed embryo transfers (ET) is recommended.

If less than 20 oocytes are retrieved, patients are instructed to start an intensive luteal support with estradiol and progesterone, the day following OPU, and are re-evaluated 3 days after oocyte retrieval (day of ET) for signs of early moderate OHSS (ultrasonographic signs of ascites as reflected by the appearance of fluid surrounding the uterus/ovaries, and/or Hct levels >40 % for the degree of haemoconcentration). The intensive luteal support includes 4 mg daily E2 valerate per os (Progynova; Schering), combined with either 50 mg progesterone IM (Gestone, Ferring- Lapidot, Israel) daily, 400 mg micronized progesterone vaginal tablets (Endometrin, Ferring-Lapidot, Israel) in two divided doses, 900 mg micronized progesterone soft gel vaginal capsules (Utrogestan, Besins, Iscovesco, C.T.S., Petach Tikva, Israel) in three divided doses, or 180 mg micronized progesterone vaginal gel (Crinone® 8 %, Merck Serono, Herzelia, Israel) in two divided doses.

If no early signs of OHSS developed, one embryo was transferred, and the patients are instructed to inject 1500 IU of HCG [21]. By deferring the hCG bolus by 3 days (5 days following GnRHa trigger), the corpus luteum is rescued, with an observed extremely high midluteal progesterone levels [22], reasonable pregnancy rate, with no patient developing severe OHSS. Moreover, by deferring the hCG bolus, we are actually offering the hCG to 80 % of the a priori at risk patients, who are not supposed to develop severe early OHSS, while avoiding hCG administration to the “real” 20–26 % [23, 24] patient at risk to develop severe early-OHSS.

In cases where signs of early moderate OHSS appear, the freeze all policy is recommended.

Patients not at risk to develop severe OHSS- three different modes of concomitant administration of both GnRHa and a standard bolus of hCG (5000–10,000 units) prior to oocyte retrieval were suggested, aiming to improve oocyte and embryo quantity and quality and the consequent IVF cycle outcome. Luteal support consists of progesterone only, in the forms of either 200 mg micronized progesterone vaginal tablets (Endometrin, Ferring-Lapidot, Israel) in two divided doses, 600 mg micronized progesterone soft gel vaginal capsules (Utrogestan, Besins, Iscovesco, C.T.S., Petach Tikva, Israel) in three divided doses, or 90 mg micronized progesterone vaginal gel (Crinone® 8 %, Merck Serono, Herzelia, Israel) once a day.

Standard hCG dose concomitant with GnRHa (dual trigger), 35-37 h before oocyte retrieval is offered to normal responders patients, resulting in higher number of oocytes retrieved [25], matured oocytes [25, 26] and number of embryos cryopreserved [25, 27], with the consequent significant increase in implantation [23], clinical pregnancy and live-birth rates [25] and the number of patients who received at least one embryo of excellent quality [27], as compared with the hCG-only trigger group [25–27].

GnRHa 40 h and standard hCG added 34 h prior to OPU (double trigger), respectively are offered to two group of patients demonstrating abnormal final follicular maturation despite normal response to COH, those with low (<50 %) number of oocytes retrieved per number of dominant follicles > 14 mm in diameter on day of hCG administration [28] and those with low proportion of mature/ metaphase-II (MII) oocytes (<66 %) per number oocytes retrieved [29]. In both groups, following the double trigger, patients had significantly higher number of oocytes retrieved, higher proportions of the number of oocytes retrieved to the number of follicles >10 mm and >14 mm in diameter on day of hCG administration, higher number of MII oocytes and proportion of MII oocytes per number of oocytes retrieved, with the consequent significantly increased number of top-quality embryos, as compared to the hCG-only trigger cycles [28, 29]. This regimen was based on the assumption, that by prolonging the time between ovulation triggering and OPU and the GnRHa trigger with the consequent simultaneous induction of an FSH surge, the “double trigger” could overcome any existing impairments in granulosa cell function, oocyte meiotic maturation or cumulus expansion [30].

Standard hCG dose concomitant with GnRHa (dual trigger), 34 h before oocyte retrieval should be offered to poor responders patients. One of the major unnoticed concern in poor responders is the observed high prevalence of premature luteinization\ovulation [31, 32]. This may be overcome by early triggering of final follicular maturation- while approaching a follicular size of 15–16 mm, and by shortening the duration between the trigger and OPU. However, since shortening the interval between hCG priming and oocyte retrieval may decrease the percentage of mature oocytes [33], dual trigger (hCG and GnRHa) administered 34 h prior to OPU will provide the additional improvement in the number of oocytes retrieved to the number of follicles >10 mm, and the proportion of mature oocytes should be implemented [28, 29].

## Conclusion

In the present opinion paper we suggested and discussed a simplified approach to COH protocol, which combines the benefits of the ultrashort flare GnRHa/GnRHant protocol and the personalized tailored mode and timing of triggering final follicular maturation in IVF patients. Further studies are required to support this new concept prior to its universal implementation to IVF practice.

## Abbreviations

COCS: combined oral contraceptives
COH: controlled ovarian hyperstimulation
ET: embryo transfer
GnRHa: GnRH agonist
hCG: human chorionic gonadotropin
IVF: in vitro fertilization
OHSS: ovarian hyperstimulation syndrome
